# Effect of initial infusion rates of fluid resuscitation on outcomes in patients with septic shock: a historical cohort study

**DOI:** 10.1186/s13054-020-2819-5

**Published:** 2020-04-07

**Authors:** Bo Hu, Joy C. Y. Chen, Yue Dong, Ryan D. Frank, Melissa Passe, Erica Portner, Zhiyong Peng, Kianoush Kashani

**Affiliations:** 1 Division of Pulmonary and Critical Care Medicine, Department of Medicine, Mayo Clinic, 200 First Street SW, Rochester, MN 55905 USA; 2 Department of Critical Care Medicine, Zhongnan Hospital of Wuhan University, 169 East Lake Road, PO Box 430071, Wuhan, Hubei China; 3 Department of Internal Medicine, Mayo Clinic, Rochester, MN USA; 4 Department of Anesthesiology and Perioperative Medicine, Mayo Clinic, Rochester, MN USA; 5 Division of Biomedical Statistics and Informatics, Mayo Clinic, Rochester, MN USA; 6 Department of Anesthesia Clinical Research Unit, Mayo Clinic, Rochester, MN USA; 7 Division of Nephrology and Hypertension, Department of Medicine, Mayo Clinic, 200 First Street SW, Rochester, MN 55905 USA

**Keywords:** Fluid resuscitation rate, Septic shock, Shock reversal, Vasopressor, Lactate clearance

## Abstract

**Background:**

Fluid resuscitation has become the cornerstone of early septic shock management, but the optimal fluid rate is still not well studied. The goal of this investigation is to examine the relationship between fluid resuscitation rate and septic shock resolution.

**Method:**

We retrospectively studied adult (≥ 18 years) patients with septic shock, defined based on sepsis III definition, from January 1, 2006, through May 31, 2018, in the medical intensive care unit (MICU) of Mayo Clinic Rochester. The fluid resuscitation time was defined as the time required to infuse the initial fluid bolus of 30 ml/kg, based on the recommendations of the 2016 surviving sepsis campaign. The cohort was divided into four groups based on the average fluid rate (group 1 ≥ 0.5, group 2 0.25–0.49, group 3 0.17–0.24, and group 4 < 0.17 ml/kg/min). The primary outcome was the time to shock reversal. Multivariable regression analyses were conducted to account for potential confounders.

**Result:**

A total of 1052 patients met eligibility criteria and were included in the analysis. The time-to-shock reversal was significantly different among the groups (*P* < .001). Patients in group 1 who received fluid resuscitation at a faster rate had a shorter time to shock reversal (HR = 0.78; 95% CI 0.66–0.91; *P* = .01) when compared with group 4 with a median (IQR) time-to-shock reversal of 1.7 (1.5, 2.0) vs. 2.8 (2.6, 3.3) days, respectively. Using 0.25 ml/kg/min as cutoff, the higher fluid infusion rate was associated with a shorter time to shock reversal (HR = 1.22; 95% CI 1.06–1.41; *P* = .004) and with decreased odds of 28-day mortality (HR = 0.71; 95% CI 0.60–0.85; *P* < .001).

**Conclusion:**

In septic shock patients, initial fluid resuscitation rate of 0.25–0.50 ml/kg/min (i.e., completion of the initial 30 ml/kg IV fluid resuscitation within the first 2 h), may be associated with early shock reversal and lower 28-day mortality compared with slower rates of infusion.

## The potential impact of this research

In this study, we assess the impact of the initial fluid replacement rate on the outcome of patients with septic shock managed based on Surviving Sepsis Campaign (SSC) guidelines. We found among septic shock patients the minimum initial fluid resuscitation rate of 0.25–0.50 ml/kg/min (i.e., completion of the initial 30 ml/kg IV fluid resuscitation within the first 2 h) is associated with a shorter time to shock reversal and improved patient outcome.

## Introduction

Septic shock refers to sepsis with cardiovascular dysfunction. It is prevalent and is associated with a high rate of mortality [1, 2]. It is characterized by systemic vasodilation and increased vascular permeability [2–4]. These changes result in impaired microcirculatory blood flow and reduced tissue perfusion [5]. The fluid resuscitation for septic shock can restore perfusion before the onset of irreversible tissue damage [4] and prevent cardiovascular collapse and death [6, 7], and, hence, lower mortality [8]. Therefore, appropriate fluid resuscitation within the first 3 h of shock state is strongly recommended by the Surviving Sepsis Campaign (SSC) guidelines [9] as the cornerstone of septic shock treatment [10].

The challenge remains to identify the optimal fluid resuscitation strategy. While the standard of practice is the use of boluses of intravascular (IV) fluid for resuscitation [6, 9], a few trials have shown increased mortality with fluid resuscitation [11, 12]. Also, three recent multi-center randomized controlled trials [13–15] and a follow-up meta-analysis [16] showed that early goal-directed therapy (EGDT) bundles in comparison with usual care were not associated with improved outcomes in septic shock. Additionally, fluid boluses could lead to a positive fluid balance and excess fluid in the interstitial space [17, 18], resulting in tissue edema, decreased oxygen delivery, and increased mortality [19–21].

These controversies surrounding the optimal strategies of initial bolus-fluid administration during septic shock resuscitation make searching for the optimal dose, type, and rate of fluid resuscitation in septic shock a research priority [22]. The current guidelines recommend at least 30 ml/kg of intravenous crystalloid fluid to be given within the first 3 h of resuscitation [9, 23], but the influence may be different based on the time to attainment of this target in fluid resuscitation. So the goal of this study is to examine the relationship between initial fluid resuscitation rate and septic shock resolution in septic shock patients.

## Methods

### Participants

This is a retrospective cohort study of adult (≥ 18 years of age) medical intensive care unit (MICU) patients in Mayo Clinic, Rochester, MN, from January 1, 2006, through May 31, 2018, who had a diagnosis of septic shock and underwent resuscitation with IV fluids. Using hospital electronic health records (EHR), we screened MICU patients for eligibility. We identified septic shock patients who received fluid resuscitation > 30 ml/kg within the first 24 h and excluded patients with other types of shock [hypovolemic shock, cardiogenic shock, obstructive shock based on the International Classification of Diseases-10 (ICD-10) code of discharge diagnosis], those without Minnesota research authorization, vulnerable adults, prisoners, individuals with known pregnancy at the time of index admission, and patients who stayed in the MICU for < 48 h. The study was reviewed and approved by the Institutional Review Board (#18-008349) at Mayo Clinic, Rochester. Informed consent was waived for patients with Minnesota research authorization due to the minimal risk nature of the study.

### Definitions

Sepsis was defined as an increase in the Sequential [Sepsis-related] Organ Failure Assessment (SOFA) score of 2 points or more, which caused by presumed or confirmed infection (Sepsis-3) [2]. To minimize the effect of temporal changes in the care of patients with sepsis during the study period and to confirm the accuracy of the included patients, the septic shock cases had to meet all three following criteria (1) diagnosis of septic shock based on ICD-10 code of discharge diagnosis, (2) criteria of sepsis described by Sepsis-3, and (3) mean arterial pressure (MAP) < 65 mmHg with vasopressor use and serum lactate level > 2 mmol/l along with an antibiotic prescription.

To minimize the effect of pre-hospital fluid resuscitation, we only included patients whose first recorded mean arterial pressure of < 65 mmHg occurred in MICU, and there was no record of vasopressor utilization prior to MICU admission. Time zero (*T*_0_) was defined as the first time that MAP was < 65 mmHg or serum lactate was > 2 mmol/l. Shock reversal time (*T*_r_) was defined as the time in which MAP was > 65 mmHg without vasopressors and lactate < 2 mmol/l. Time to shock reversal was calculated as the duration between *T*_r_ and *T*_0._

**Fig. 1:**
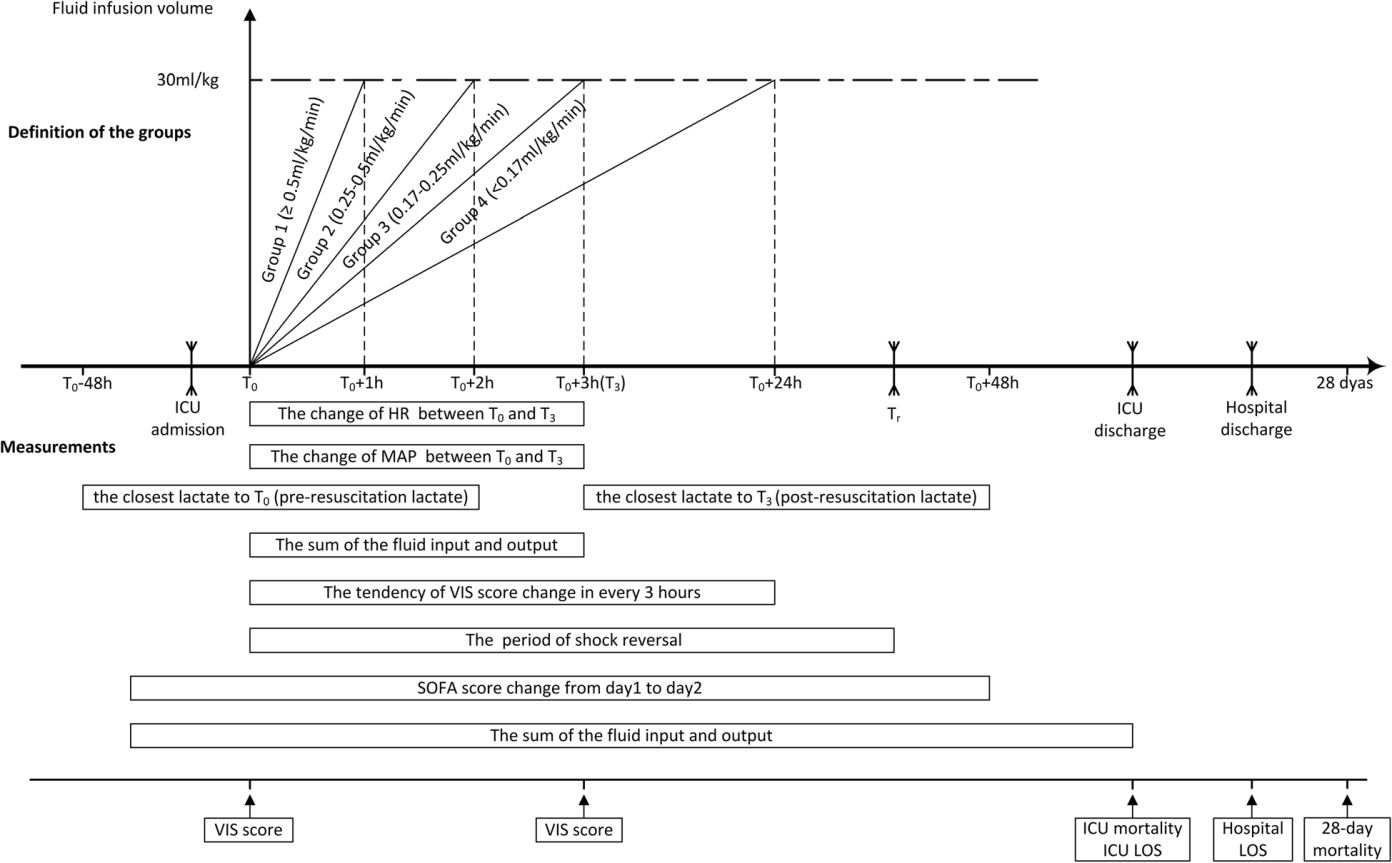
Schematic representation of the study protocol. Time zero (*T*_0_) was the starting point of septic shock fluid resuscitation and defined as the first time that MAP < 65 mmHg or serum lactate > 2 mmol/l during the ICU stay. According to the different range of initial fluid resuscitation rate (equal to the slope in the graph), the cohort was divided into four groups: group 1 (≥ 0.5 ml/kg/min), group 2 (0.25–0.5 ml/kg/min), group 3 (0.17–0.25 ml/kg/min), and group 4 (< 0.17 ml/kg/min). The “” on the timeline marked ICU admission, shock reversal time (*T*_r_), and ICU and hospital discharge; shock reversal time (*T*_r_), ICU discharge, and hospital discharge must be after *T*_0_ but had no fixed time relationship with *T*_0_. The main variables and measurements of the study are shown in the bottom half of the figure. The period of shock reversal was defined as the duration between *T*_r_ and *T*_0_ (abbreviation: ICU = intensive care unit; *T*_0_ = time zero; *T*_3_ = 3 h after *T*_0_; *T*_r_ = shock reversal time; VIS = Vasoactive-Inotropic Score; LOS = length of stay)

The initial fluid resuscitation rate (ml/kg/min) was calculated as the volume of 30 ml/kg of the actual body weight on admission divided by the time (min) to complete. Although the literature in support of the use of 30 ml/kg of crystalloid for initial volume resuscitation among septic shock patients is scarce, it is considered as one of the major recommendations by SSC_2016_. Therefore, we chose 30 ml/kg of crystalloid as a cutoff for the inclusion of patients in our study. In a recent study, investigators demonstrated that failure to deliver 30 ml/kg within 3 h of diagnosis of sepsis was associated with increased odds of in-hospital mortality, irrespective of other comorbidities [24]. Patients were categorized into four groups based on the resuscitation time: ≤ 1 h, 1.1–2 h, 2.1–3 h, and > 3 h for groups 1 to 4, respectively. The corresponding fluid rate for the groups described above were ≥ 0.5, 0.25–0.49, 0.17–0.24, and < 0.17 ml/kg/min, respectively (Fig. 1).

Pre-resuscitation lactate was defined as the lactate level closest (from 48 h before *T*_0_ to 2 h after *T*_0_) to *T*_0_, post-resuscitation lactate was defined as the lactate level closest to *T*_3_ (3 h after *T*_0_), and lactate clearance was determined by its decline between pre- and post-resuscitation lactate levels. The doses of the vasopressors were described by Vasoactive-Inotropic Score (VIS; [VIS = dopamine dose (mcg kg^−1^ min^−1^) + dobutamine dose (mcg kg^−1^ min^−1^) + 100 × epinephrine dose (mcg kg^−1^ min^−1^) + 10,000 × vasopressin dose (units kg^−1^ min^−1^) + 100 × norepinephrine dose (mcg kg^−1^ min^−1^) + 100 × phenylephrine dose (mcg kg^−1^ min^−1^)]) [25]. Fluid balance was defined as the difference of the fluid intake and output and was adjusted based on hospital admission weight. Acute Physiology And Chronic Health Evaluation (APACHE) III and SOFA scores were automatically calculated. Charlson comorbidity index (CCI) was determined at hospital admission.

### Variables and outcomes

Baseline variables including patient demographics, hospital admission weight, hemodynamic variables, sites of infection, APACHE III and SOFA scores, and CCI were collected from the Multidisciplinary Epidemiology and Translational Research in Intensive Care (METRIC) DataMart [26]. The primary outcome was the time to shock reversal. Secondary outcomes included lactate clearance, weight-adjusted fluid balance in the first 3 h of resuscitation and throughout MICU stay, weight-adjusted fluid infusion between *T*_3_ and MICU discharge, timing of vasopressor initiation, temporal trends of VIS in the first 24 h calculated every 3 h, MAP and heart rate changes within the first 3 h of resuscitation, need and length of mechanical ventilation, SOFA day 1 to day 2 score changes, time to alive discharge from MICU and hospital, and finally MICU, hospital, and 28-day mortality (Fig. 1).

### Statistical analysis

We summarized the data using frequencies and percentages for categorical variables and medians and interquartile ranges for continuous variables. We also compared data distributions across fluid resuscitation rate groups using chi-square and Kruskal-Wallis tests for categorical and continuous data, respectively.

Associations between fluid resuscitation rate and outcomes were analyzed using the univariable and multivariable models to adjust for age, sex, race, weight, CCI, APACHE III, and SOFA scores. We used logistic regression for binary outcomes (i.e., hospital and 28-day mortality) and linear regression for continuous outcomes (i.e., lactate clearance, fluid balance). The time to shock reversal and alive discharge from MICU, hospital, and 28 days were analyzed using Cox proportional hazards regression models. Associations between fluid resuscitation rate and VIS at different time points were analyzed using a multivariable generalized estimated equation model to adjust for the same variables listed previously. The median and interquartile range of VIS was plotted at hours 0, 6, 12, and 18 by fluid resuscitation completion time. All analyses were performed using SAS version 9.4 (SAS Institute Inc., Cary, NC). A 2-sided *P* value < 0.05 was determined to be significant.

## Result

**Fig. 2:**
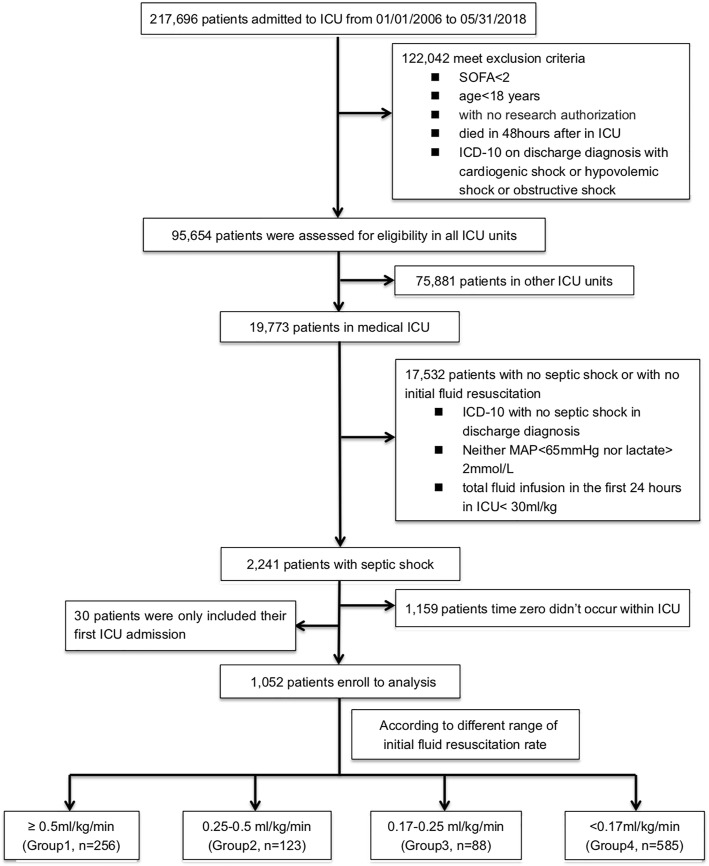
The CONSORT flow diagram of patient enrollment (abbreviation: ICU = intensive care unit; SOFA = Sequential [Sepsis-related] Organ Failure Assessment; ICD = International Classification of Diseases; MAP = mean arterial pressure)

We screened 217,696 patients who were admitted to ICU from January 01, 2006, to May 31, 2018 (Fig. 2), and 1052 individuals who met all eligibility criteria entered the final analyses. Of those, 256 (24.3%) were in group 1, 123 (11.7%) in group 2, 88 (8.4%) in group 3, and 585 (55.6%) in group 4. Baseline characteristics for each category are presented in Table 1. Supplementary Figures 1A, B, and C show the average fluid intake, output, and balance, respectively, among all participants during the first 7 days of ICU admission. The four groups were similar with respect to demographic characteristics, comorbid conditions, and severity of illness. Patients with slower fluid resuscitation rates were heavier at baseline.

**Table 1:** Clinical demographics and baseline characteristics by initial fluid resuscitation rate (ml/kg/min)

Variables	Fluid replacement rate; ml/kg/min				*P* value
	≥ 0. 5, *N* = 256	0.25–0.49, *N* = 123	0.17–0.24, *N* = 88	< 0.17, *N* = 585	
Age; year, median (IQR)	66 (55, 78)	68 (59, 80)	70 (53, 79)	67 (56, 77)	.5^†^
Male sex; *N* (%)	126 (49%)	65 (53%)	40 (46%)	296 (51%)	.7^‡^
White race; *N* (%)	242 (95%)	114 (93%)	79 (90%)	526 (90%)	.2^‡^
Hospital admission weight; kg, median (IQR)	76 (63, 90)	81 (64, 100)	80 (67, 103)	84 (69, 99)	< .001^†^
Charlson comorbidity index; median (IQR)	5 (3, 8)	6 (4, 8)	6 (3, 8)	6 (4, 8)	.9^†^
APACHE III score (*T*_*0*_); median (IQR)	82 (64, 104)	83 (69, 106)	86.5 (73, 107)	86 (70, 103)	.3^†^
SOFA score (*T*_0_); median (IQR)	8 (6, 11)	9 (7, 12)	9.0 (6.5, 11.0)	9 (6, 12)	.4^†^
Infection source					
Blood	73 (29%)	32 (26%)	25 (28%)	155 (26%)	.9
Respiratory	95 (37%)	38 (31%)	37 (43%)	274 (47%)	.002
Abdominal/GI	44 (17%)	13 (11%)	10 (11%)	78 (13%)	.2
Urinary	55 (21%)	28 (23%)	32 (37%)	135 (23%)	.02
Soft tissue and bone	24 (9%)	11 (9%)	13 (15%)	41 (7%)	.08
Other	23 (9%)	13 (11%)	12 (14%)	54 (9%)	.5
Heart rate (*T*_0_), bpm; median (IQR)	101 (88, 116)	100 (83, 113)	105 (88, 117)	98 (83, 111)	.007
Mean arterial pressure (*T*_0_), mmHg; median (IQR)	64 (57, 73)	60 (54, 68)	65 (57, 75)	66 (59, 76)	< .001
VIS (*T*_0_); median (IQR)	1.6 (0.5, 10.1)	1.5 (0.6, 12.8)	1.7 (0.7, 7.2)	1.7 (0.6, 10.6)	.9

Numbers indicate *N* (%) unless otherwise noted

^†^Kruskal-Wallis

^‡^Chi-square

**Fig. 3:**
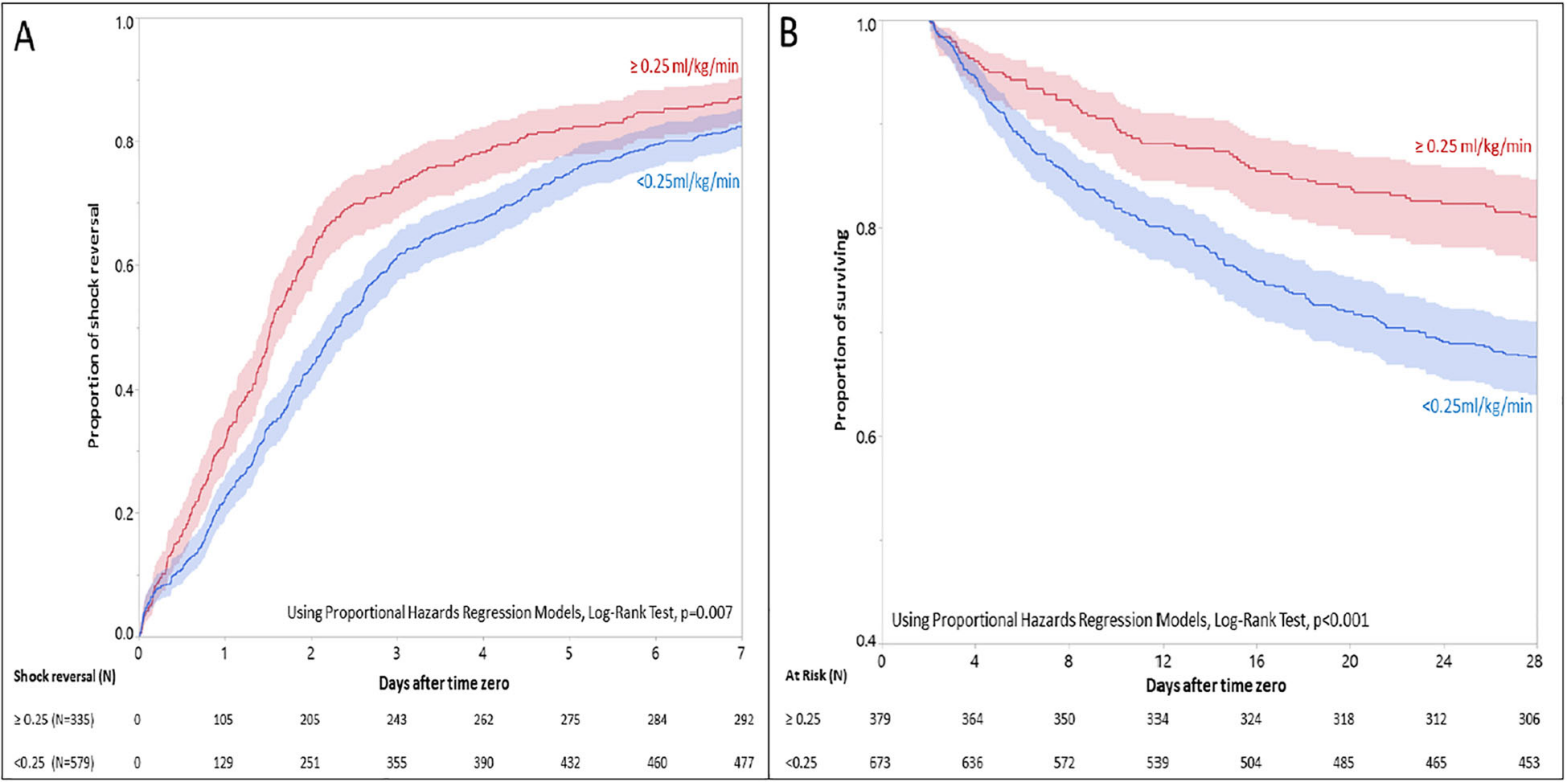
The cumulative proportion of shock reversal and survival analysis with different initial fluid resuscitation rates (≥ 0.25 ml/kg/min vs. < 0.25 ml/kg/min). The shade indicates the confidence interval. **a** The cumulative proportion of shock reversal. Using Cox-model (additionally adjusted for the effects of age, gender, white race, weight, CCI, APACHE III, and SOFA), patients with ≥ 0.25 ml/kg/min rate had higher proportion of shock reversal (HR = 1.22; 95% CI 1.06–1.41; *P* = .007), with shorter median time (IQR) to shock reversal [1.5 (1.4, 1.7) vs. 2.3 (2.1, 2.6) days] for patients with < 0.25 ml/kg/min rate. **b** Survival analysis. Using Cox-model (additionally adjusted for the effects of age, gender, white race, weight, CCI, APACHE III, and SOFA), patients with ≥ 0.25 ml/kg/min rate had higher proportion of surviving (HR = 0.71; 95% CI 0.60–0.85; *P* < .001) for patients with < 0.25 ml/kg/min rate

Among the groups, 91, 84, 83, and 87% achieved shock reversal in groups 1 to 4, respectively (*P* = .2). However, the time to shock reversal was significantly different (*P* < .001) among the groups. In multivariable analyses after adjustment for a priori independent variables, patients in group 1 achieved shock reversal faster compared to patients in group 4 (HR = 0.78; 95% CI 0.66–0.91) with a median (IQR) time to shock reversal of 1.7 (1.5, 2.0) vs. 2.8 (2.6, 3.3) days, respectively. When we used the year of admission in the multivariable models, it was not a significant predictor of outcomes (results are not shown). Time to shock reversal was not different when we compared groups 2 and 3 with group 1 (Table 2). Using 0.25 ml/kg/min as threshold, more patients with a higher fluid rate (≥ 0.25 ml/kg/min) achieved shock reversal (HR = 1.22; 95% CI 1.06–1.41; *P* = .004) with a significant shorter median (IQR) time to shock reversal [1.5 (1.4, 1.7) vs. 2.3 (2.1, 2.6) days] compared to patients with a lower fluid rate (< 0.25 ml/kg/min) (Fig. 3a).

**Table 2:** Comparison of outcomes by four different initial fluid resuscitation rate (ml/kg/min)

Variables	Fluid replacement rate; ml/kg/min				
	≥ 0.5, *N* = 256	0.25–0.49, *N* = 123	0.17–0.24, *N* = 88	< 0.17, *N* = 585	*P* value
Time to shock reversal; days					< .001^‡^
*N*	232	103	73	506	
Median (Q1, Q3)	1.6 (0.9, 3.3)	1.4 (0.6, 3.2)	2.0 (0.9, 4.6)	2.4 (1.2, 5.1)	
Median survival	1.72 (1.52, 2.01)	1.87 (1.32, 2.62)	2.58 (2.02, 4.40)	2.80 (2.57, 3.26)	.004^§^
Adjusted hazard ratio^a^	1.00 (ref)	0.96 (0.76, 1.21)	0.84 (0.65, 1.10)	0.78 (0.66, 0.91) ^**^	.01^¶^
Lactate clearance; mg/dl					< .001^‡^
*N*	207	97	62	400	
Median (Q1, Q3)	0.5 (− 0.1, 1.3)	0.4 (− 0.05, 1.2)	0.6 (− 0.04, 1.2)	0.2 (− 0.4, 0.8)	
Adjusted mean estimate ^c^	0.00 (ref)	0.05 (− 0.27, 0.38)	0.13 (− 0.25, 0.52)	− 0.45 (− 0.68, − 0.23)***	< .001^¶^
MAP change in the first 3 h; mmHg					< .001 ^‡^
*N*	254	120	79	508	
Median (Q1, Q3)	4.3 (− 3.0, 11.5)	4.0 (− 4.5, 11.5)	− 0.5 (− 10.5, 7.0)	0.5 (− 5.5, 7.0)	
Adjusted mean estimate^c^	0.00 (ref)	− 1.14 (− 4.25, 1.96)	− 3.55 (− 7.17, 0.07)	− 2.93 (− 5.10, − 0.75)**	.04^¶^
HR change in the first 3 h; bpm					.2^‡^
*N*	255	120	82	545	
Median (Q1, Q3)	− 2.0 (− 7.5, 2.5)	− 2.3 (− 8.0, 4.3)	− 2.5 (− 9.5, 0.5)	− 1.5 (− 7.5, 3.5)	
Adjusted mean estimate^c^	0.00 (ref)	0.50 (− 2.06, 3.07)	− 1.41 (− 4.37, 1.54)	1.63 (− 0.15, 3.41)	.08^¶^
Fluid balance at *T*_3_; ml/kg	59 (45, 79)	44 (35, 60)	33 (31, 41)	7 (1, 15)	< .001^‡^
Adjusted mean estimate^c^	0.00 (ref)	14.61 (10.63, 18.59)***	28.73 (24.23, 33.22)***	57.08 (54.34, 59.82)***	< .001^¶^
MICU fluid balance; ml/kg	76 (48, 136)	71 (40, 125)	66 (24, 146)	50 (16, 110)	< .001^‡^
Adjusted mean estimate^c^	0.00 (ref)	− 10 (− 34, 14)	− 6 (− 33, 21)	13 (− 4, 29)	.1^¶^
Fluid infusion between *T*_3_ and MICU discharge; ml/kg	107 (50, 264)	124 (49, 223)	158 (62, 381)	168 (86, 345)	< .001^‡^
Adjusted mean estimate^c^	0.00 (ref)	− 54 (− 102, − 5)*	37 (− 18, 91)	59 (27, 90)***	< .001^¶^
Duration between first vasopressor using and *T*_0_; hours					< .001^‡^
*N*	233	102	75	502	
Median (Q1, Q3)	2.0 (0.5, 4.7)	2.3 (1.3, 3.9)	3.7 (1.4, 6.7)	7.0 (2.2, 17.7)	
Adjusted mean estimate^c^	0.00 (ref)	− 3.8 (− 12.5, 5.0)	− 2.0 (− 11.7, 7.8)	10.3 (4.6, 15.9)***	< .001^¶^
SOFA change (day 2–day 1)					< .001^‡^
*N*	240	114	80	561	
Median (Q1, Q3)	− 3 (− 5, − 1)	− 2 (− 5, − 1)	− 2 (− 3.5, − 0.5)	− 2 (− 4, 0)	
Adjusted mean estimate^c^	0.00 (ref)	− 0.09 (− 0.71, 0.53)	0.85 (0.15, 1.55)*	0.94 (0.52, 1.36)***	< .001^¶^
Mechanical ventilation; *N* (%)	134 (52%)	68 (55%)	55 (63%)	383 (66%)	.002^†^
Adjusted odds ratio^b^	1.00 (ref)	1.04 (0.63, 1.75)	1.56 (0.87, 2.83)	1.91 (1.34, 2.73)***	.001^¶^
Mechanical ventilation duration; days					.002^‡^
*N*	134	68	55	383	
Median (Q1, Q3)	3 (1, 7)	2 (1, 6)	5 (2, 9)	4 (2, 8)	
Adjusted mean estimate^c^	0.00 (ref)	− 14.95 (− 66.76, 36.87)	43.67 (− 11.93, 99.27)	45.07 (9.60, 80.54)*	.01^¶^
Discharge alive from MICU	238 (93%)	112 (91%)	81 (92%)	481 (82%)	< .001^†^
Median survival; days	2.89 (2.64, 3.19)	3.09 (2.42, 3.86)	4.06 (3.12, 5.99)	4.90 (4.51, 5.67)	< .001^§^
Adjusted hazard ratio^a^	1.00 (ref)	0.95 (0.75, 1.19)	0.74 (0.57, 0.95)*	0.58 (0.49, 0.68)***	< .001^¶^
Discharge alive from hospital	225 (88%)	93 (76%)	68 (77%)	415 (71%)	< .001^†^
Median survival; days	10.09 (8.51, 11.80)	12.51 (9.77, 15.03)	16.81 (13.66, 21.01)	17.69 (15.45, 19.96)	< .001^§^
Adjusted hazard ratio^a^	1.00 (ref)	0.84 (0.66, 1.08)	0.58 (0.44, 0.76)***	0.53 (0.45, 0.63)***	< .001^¶^
MICU mortality; *N* (%)	18 (7%)	11 (9%)	7 (8%)	104 (18%)	< .001^†^
Adjusted odds ratio^b^	1.00 (ref)	1.34 (0.59, 3.03)	1.12 (0.44, 2.87)	3.40 (1.95, 5.93)***	< .001^¶^
Hospital mortality; *N* (%)	31 (12%)	30 (24%)	20 (23%)	170 (29%)	< .001^†^
Adjusted odds ratio^b^	1.00 (ref)	2.53 (1.41, 4.52)**	2.23 (1.17, 4.27)*	3.51 (2.27, 5.44)***	< .001^¶^
28-day mortality; *N* (%)	41 (16%)	32 (26%)	21 (24%)	199 (34%)	< .001^†^
Adjusted odds ratio^b^	1.00 (ref)	1.97 (1.12, 3.45)*	1.86 (0.99, 3.51)	3.19 (2.13, 4.78)***	< .001^¶^

Numbers indicate *N* (%) and median (Q1, Q3) unless otherwise noted

Unadjusted models contain just fluid resuscitation rate adjusted models are additionally adjusted for the effects of age, gender, white race, weight, CCI, APACHE III, and SOFA

Compared to ≥ 0. 5 ml/kg/min group, *P value < 0.05, **P value < 0.01, ***P value < 0.001

^†^Chi-square

^‡^Kruskal-Wallis

^§^Log-rank test

^¶^Type 3 Wald test

^a^Analyzed using proportional hazards regression models

^b^Analyzed using logistic regression models

^c^Analyzed using linear regression models

### Secondary outcomes

The time of pre-resuscitation reported lactate was 0 (− 3.6, 1.1) h from *T*_0_, and the time of post-resuscitation reported lactate was 3.8 (1.5, 10.6) h after *T*_3_. The lactate clearance during initial fluid resuscitation was significantly different among groups (*P* < .001); group 4 had less lactate reduction compared to group 1 (0.2 vs. 0.5 mg/dl; *P* < .001). Group 4 also had minimal change in MAP at 3 h, compared to group 1, who had a median increase of 4.3 mmHg (*P* = .04). A lower initial fluid resuscitation rate was associated with a later vasopressor use after septic shock onset (*P* < .001). A higher initial fluid resuscitation rate was associated with a higher mean fluid balance at 3 h (*P* < .001), but not with the mean fluid balance for the rest of the MICU stay (*P* = .1). The volume of infused fluid from *T*_3_ to the MICU discharge was significantly higher in group 4 compared to groups 1 (168 vs. 107 ml/kg, *P* < .001) and 2 (168 vs. 124 ml/kg, *P* < .001). Relative to group 4, fewer patients in group 1 required mechanical ventilation (OR = 1.91; 95% CI = 1.34, 2.73; *P* = .001), and for the ones who needed mechanical ventilation, the duration was shorter (mean = 1.88; 95% CI = 0.40, 3.36 days; *P* = .01). Group 1 also had a more significant decline in SOFA score compared to groups 3 (− 3 vs. − 2; *P* < .05) and 4 (− 3 vs. − 2, *P* < .001) (Table 2).

The proportions of alive discharges from ICU and hospital (*P* < .001 for both) significantly differed among the groups. Similarly, MICU (*P* < .001) and hospital durations (*P* < .001) were different. Compared to group 4, group 1 had a shorter MICU (2.9 vs.4.9 days; *P* < .001) and hospital (10.1 vs. 17.7 days; *P* < .001) stay. The 28-day mortality was also significantly different among the groups (*P* < .001). Compared to group 4, group 1 had lower 28-day mortality (16.0% vs.34.0%; *P* < .001) (Table 2). Using 0.25 ml/kg/min as threshold for intravascular fluid replacement rate, patients with a higher fluid rate (≥ 0.25 ml/kg/min) were more likely to survive at 28 days (HR = 0.71; 95% CI 0.60–0.85; *P* < .001) compared to ones with a lower fluid rate (< 0.25 ml/kg/min) (Fig. 3b).

**Fig. 4:**
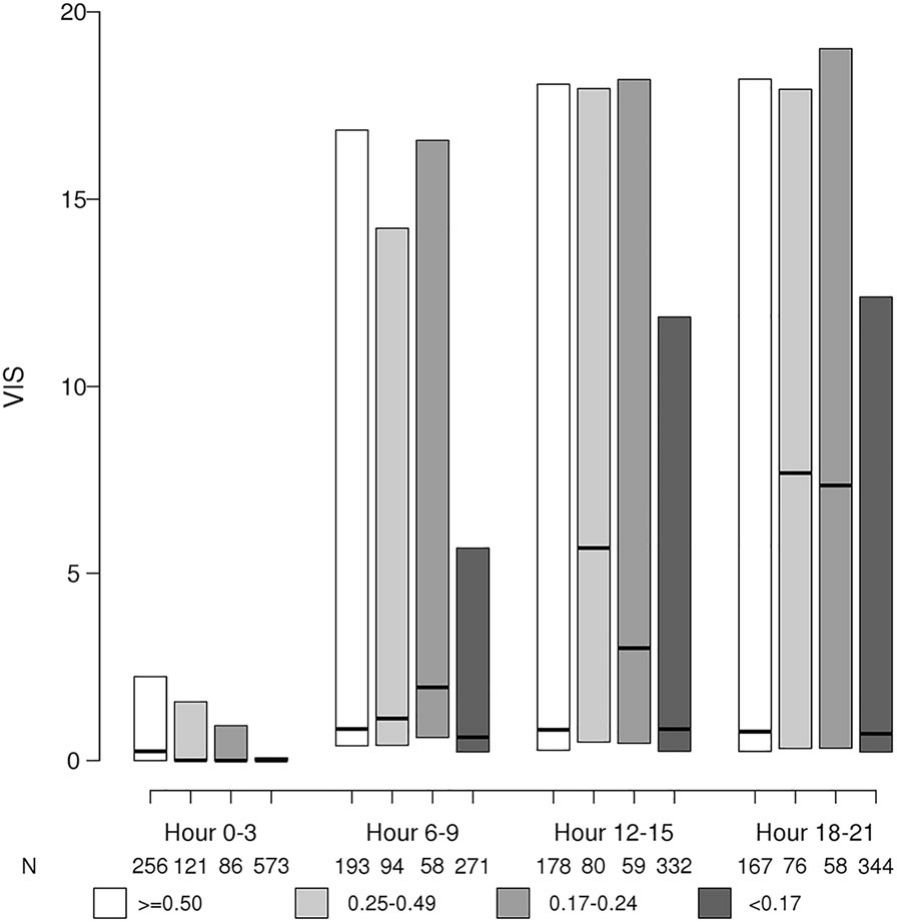
Association between fluid resuscitation rate (ml/kg/min) and VIS at variable time periods during the first 24 h of fluid resuscitation. Group < 0.17 ml/kg/min had significantly lower VIS at all time period. Compared to group < 0.17 ml/kg/min, *P value < 0.05, **P value < 0.01 (abbreviation: VIS = Vasoactive-Inotropic Score)

Table 3 and Fig. 4 show associations between fluid resuscitation rate and VIS at various time points. Fluid rate of < 0.17 ml/kg/min and increased weight were associated with lower VIS scores at all time points. Increased length of ICU stay, APACHE III, and SOFA scores were associated with increased VIS scores.

**Table 3:** Associations between fluid time and VIS by hour using univariate and multivariable GEE linear regression models

Variables	Univariate analysis^†^		Multivariable analysis^‡^	
	Mean estimate (95% CI)	*P* value	Mean estimate (95% CI)	*P* value
Fluid replacement rate; ml/kg/min		< .001		< .001
≥ 0.5	0.00 (ref)		0.00 (ref)	
0.25–0.49	0.18 (− 1.89, 2.24)		0.11 (− 1.77, 2.00)	
0.17–0.24	− 0.03 (− 2.34, 2.28)		− 0.15 (− 2.23, 1.92)	
< 0.17	− 2.62 (− 4.08, − 1.16)		− 2.41 (− 3.79, − 1.03)	
VIS time (per 1 h)	0.20 (0.15, 0.24)	< .001	0.18 (0.13, 0.22)	< .001
Age (per year)	− 0.01 (− 0.05, 0.03)	.6	− 0.03 (− 0.08, 0.02)	.2
Male sex	− 0.07 (− 1.25, 1.11)	.9	0.33 (− 0.74, 1.41)	.5
White race	− 0.73 (− 2.79, 1.34)	.5	− 0.24 (− 2.18, 1.70)	.8
Weight (per 10 kg)	− 0.5 (− 0.7, − 0.4)	< .001	− 0.5 (− 0.7, − 0.3)	< .001
Charlson comorbidity index	0.02 (− 0.19, 0.23)	.9	0.04 (− 0.18, 0.27)	.7
APACHE III score	0.09 (0.07, 0.11)	< .001	0.05 (0.01, 0.08)	.007
SOFA	0.68 (0.52, 0.84)	< .001	0.41 (0.16, 0.66)	< .001

^†^Mean estimate and *P* value are adjusted only for the row covariate

^‡^Mean estimate and *P* value are adjusted for all covariates listed in the table

## Discussion

In this retrospective analysis of MICU patients with septic shock who received fluid resuscitation, we demonstrated that a fluid resuscitation rate > 0.25 ml/kg/min was associated with a shorter time to shock reversal and lower 28-day mortality. Based on our observations, the optimal fluid rate was 0.25–0.5 ml/kg/min (equivalent to 30 ml/kg fluid administration within 2 h). Faster initial fluid resuscitation, in our study, was also associated with lower 28-day mortality. This finding is consistent with a recent prospective observational cohort study, which found that sepsis patients who received initial fluid resuscitation in > 2 h after diagnosis had increased mortality rates [27]. Vincent also suggested administration of 30 ml/kg of fluid over 3 h to be too slow for appropriate fluid resuscitation [28].

The goal of initial fluid resuscitation in septic shock is to restore intravascular volume, cardiac output, and oxygen delivery [29]. To restore intravascular volume, the rate of initial fluid infusion should be faster than the speed of fluid loss via leaky vascular endothelium. A faster initial fluid resuscitation rate could also decrease the early inflammation [30] and blood viscosity [31] to improve the microcirculation and tissue perfusion [4]. A higher fluid rate is also associated with a more significant increase in MAP and a greater reduction in lactate, a marker for tissue perfusion [32]. This finding is consistent with previous reports that showed improved microcirculation in sepsis following the early administration of fluids [33]. The improvement both in macrocirculation and microcirculation can lead to improved outcomes including less time to shock reversal, need and duration of mechanical ventilation, SOFA score, hospital, MICU stay, and 28-day mortality rates.

On the other hand, very fast fluid replacement may increase glycocalyx shedding and exacerbate vascular dysfunction. The endothelial glycocalyx layer is damaged during sepsis, which negatively impacts its barrier function [34–36]. Receiving 40–60 ml/kg IV fluid boluses in 1 h (up to 0.67–1 ml/kg/min) [11] among critically ill children led to a cardiovascular collapse in a large trial [19]. Bryne and colleagues found a rapid increase in vasopressor requirement and a significant increase in glycocalyx layer damage after initial fluid resuscitation in an ovine model of endotoxemia following fluid infusion rate of 0.67 ml/kg/min [37]. In our study, while we noted rapid fluid replacement is associated with improved outcomes of septic shock, we were not able to assess the impact of very high fluid rates (> 0.67 ml/kg/min).

We also identified an association between a lower initial fluid resuscitation rate and a lower VIS at various time points in the first 24 h of septic shock onset. This result was most likely due to later initiation of vasopressors and the impact of higher baseline weight in calculating VIS [38–40]. A higher VIS at 48 h after cardiovascular surgery was found to be associated with a longer ICU length of stay and longer ventilator days in pediatric sepsis patients [41]. In our study, group 4 with a lower fluid resuscitation rate and a lower VIS in the first 24 h had worse clinical outcomes. This discrepancy could be due to not assessing VIS beyond 24 h. Also, the relationship between VIS and patient outcomes has not been validated in sepsis.

In our study, a faster initial fluid resuscitation rate was associated with a larger positive fluid balance in the first 3 h of resuscitation, but the differences in fluid balance among the groups were not significant at MICU discharge. Treating patients with septic shock inevitably would result in initial positive fluid balance, which in the early phases of fluid resuscitation increases cardiac output in most patients [42]. In a study by Lee et al., septic shock patients who received a larger volume of fluid in the first 3 h were more likely to survive [43]. The differences in positive fluid balance among the groups resolved beyond the first 3 h because group 1 received fewer fluids after the initial resuscitation phase. Patients who receive less fluid in the first 6 h have significantly higher fluid balance in the next 7 to 72 h, in-hospital, and 28-day mortality [44]. Shen and colleagues also showed a positive fluid balance during the second, but not the first 24 h of septic shock was associated with increased mortality in sepsis [45]. Positive fluid balance during the initial resuscitation phase is associated with the improved outcome as long as the fluid balance is carefully monitored after the resuscitation targets are met.

Our study has several limitations. Due to its retrospective design, we are not able to imply any causal relationship [46]. Hence, prospective studies are required to verify our results. We included patients whose first recorded MAP of < 65 mmHg happened in MICU and had no record of vasopressor utilization prior to MICU admission. Despite this, we still could not completely eliminate the effect of potential fluid administration prior to the MICU admission. There is growing knowledge indicating that fluid resuscitation should be guided by fluid responsiveness [47]. Meanwhile, as one of the limitations of our study, we were not able to acquire fluid responsiveness data. As SSC guidelines indicate the use of 30 ml/kg to septic shock patients, we believe our results are still within recommended guidelines even though it is not based on fluid responsiveness assessment. In our institution, the administration of the second or third bolus of 30 ml/kg of fluids is only guided by the fluid responsiveness assessment. Lastly, the study period spanned 12 years, and changes in the clinical practice could have led to bias in our results.

## Conclusion

In septic shock patients, the minimum fluid resuscitation rate of 0.25–0.50 ml/kg/min (i.e., completion of the initial 30 ml/kg IV fluid resuscitation within the first 2 h) is associated with a shorter time to shock reversal and improved patient clinical outcomes. Our findings would serve as hypothesis-generating information in order to design and conduct prospective trials for validation.

## Supplementary information

**Additional file 1 Supplementary Figure 1.** Fluid assessment in the first seven days after time zero; A) fluid input, B) fluid output, C) fluid balance.

## Data Availability

The data used for this research are available from the corresponding author on reasonable request and subject to Institutional Review Board guidelines.
