# Supplementary figures and images for: Effect of initial infusion rates of fluid resuscitation on outcomes in patients with septic shock: a historical cohort study
Source: Crit Care. 2020 Apr 7;24:137. doi: 10.1186/s13054-020-2819-5 (PMC7140334; doi:10.1186/s13054-020-2819-5)

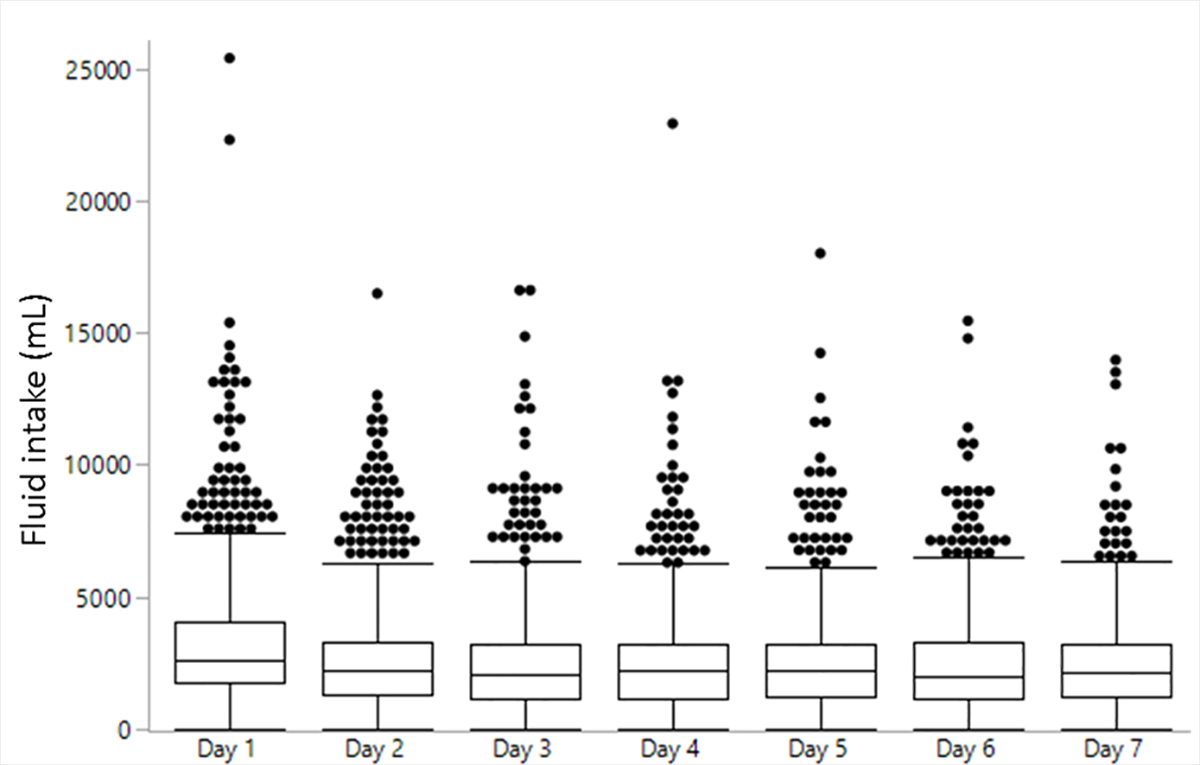

Supplement: Supplementary file 1 — Additional file 1 Supplementary Figure 1. Fluid assessment in the first seven days after time zero; A) fluid input, B) fluid output, C) fluid balance. [file 13054_2020_2819_MOESM1_ESM.zip › Supplementary Figure 1A.tif]

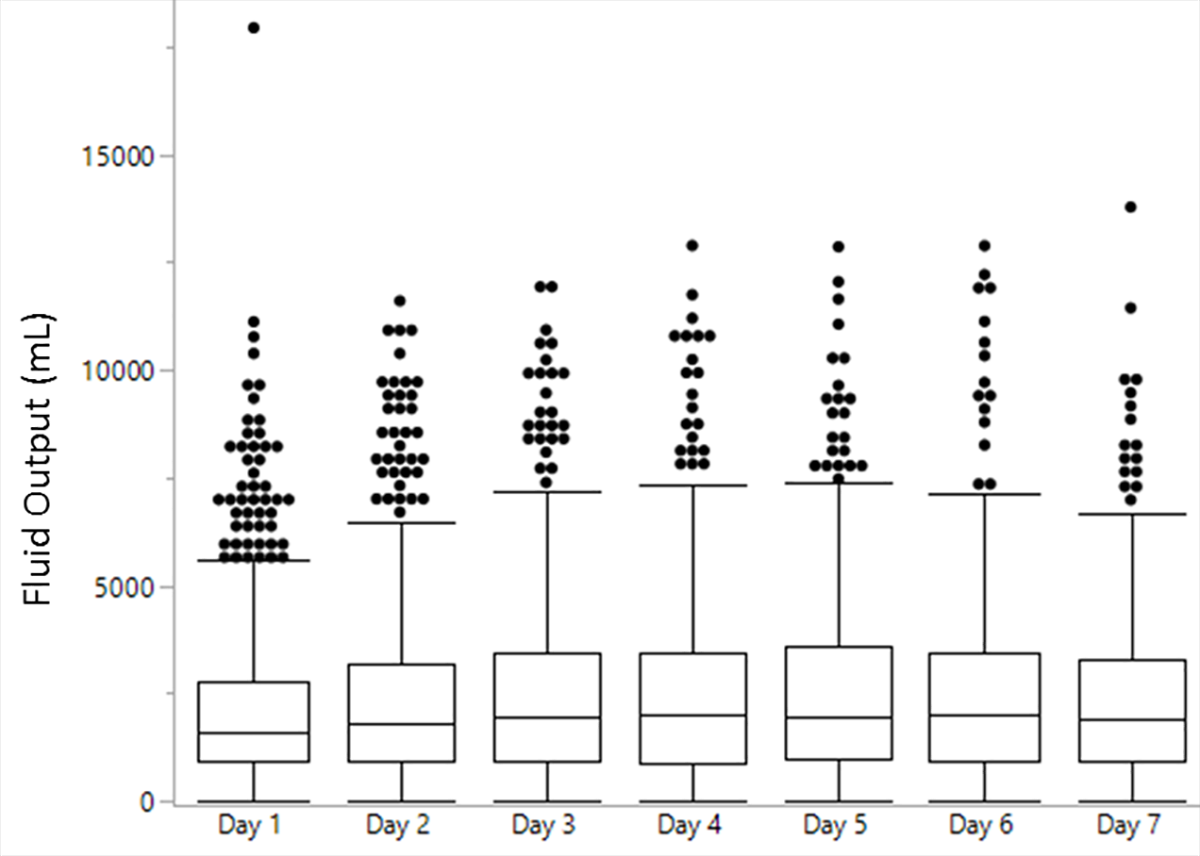

Supplement: Supplementary file 1 — Additional file 1 Supplementary Figure 1. Fluid assessment in the first seven days after time zero; A) fluid input, B) fluid output, C) fluid balance. [file 13054_2020_2819_MOESM1_ESM.zip › Supplementary Figure 1B.tif]

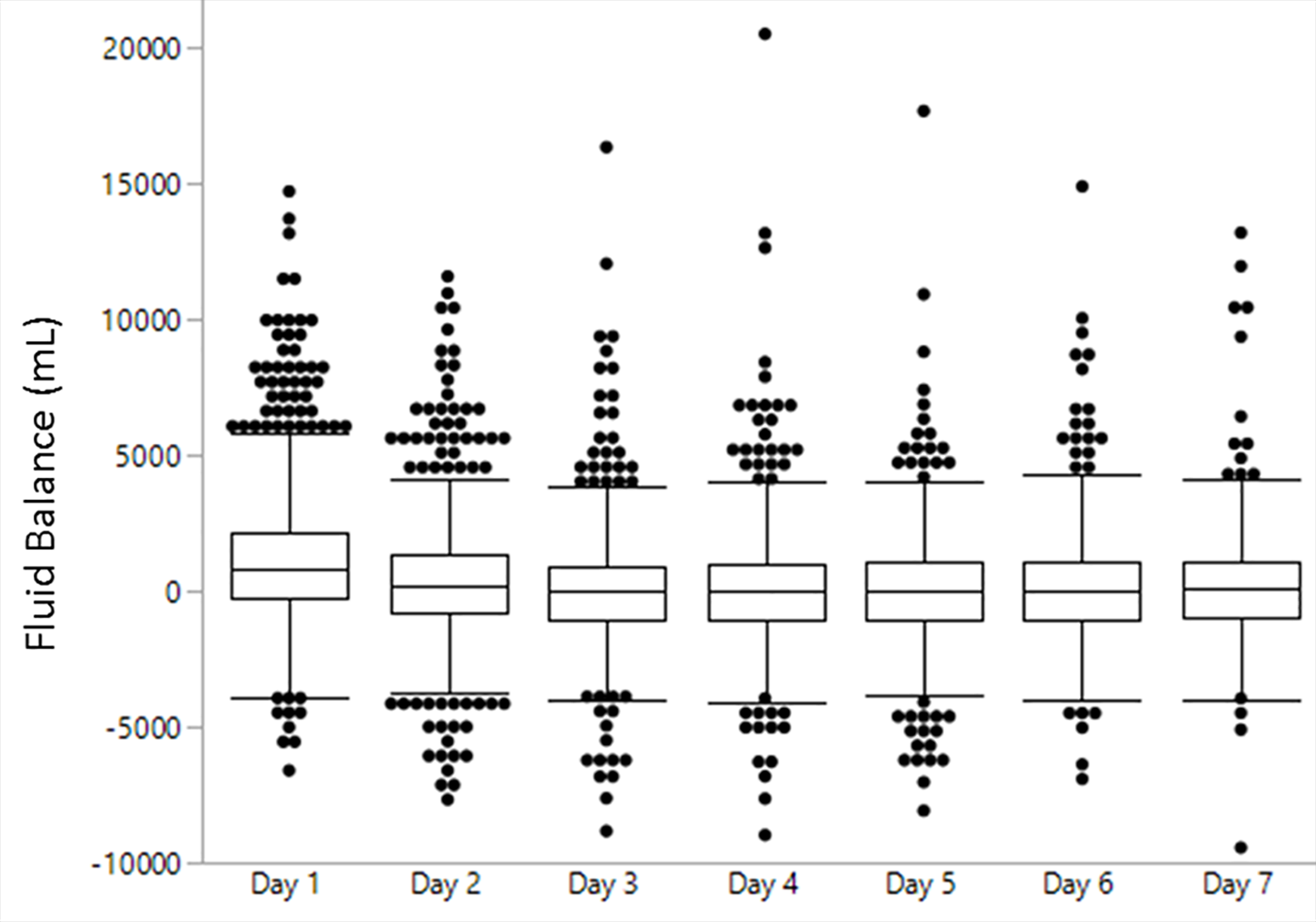

Supplement: Supplementary file 1 — Additional file 1 Supplementary Figure 1. Fluid assessment in the first seven days after time zero; A) fluid input, B) fluid output, C) fluid balance. [file 13054_2020_2819_MOESM1_ESM.zip › Supplementary Figure 1C.tif]
